# Postoperative complications in Australia and New Zealand (the REASON study)

**DOI:** 10.1186/2047-0525-2-16

**Published:** 2013-07-03

**Authors:** David A Story

**Affiliations:** 1 Centre for Anaesthesia, Perioperative and Pain Medicine, Melbourne Medical School, The University of Melbourne, Melbourne, Vic, Australia; 2 Anaesthesia, Melbourne Medical School, Level 2, Medical Building (181), The University of Melbourne, Parkville, Melbourne, Vic 3010, Australia

## Abstract

Perioperative medicine is difficult to define, and thus, its progress may be hindered until internationally accepted definitions can be established within the field. The immediate agenda for perioperative physicians should be to agree upon such definitions in order to facilitate advancements in research, audits and outcome measurements. The REASON study, looking at institutions throughout New Zealand and Australia, confirmed that postoperative complications and mortality are important areas for multicentre trials.

## The REASON Study: Methods and Hypothesis

The REASON study compared patient data from three Melbourne hospitals (Austin, Alfred and Royal Melbourne) to data from 20 institutions in all the capital cities across New Zealand and Australia, as well as smaller centres in Tasmania and rural NSW. The study was published in Anaesthesia [1] and included around 4,000 patients in total. The aim of REASON was to show that overall morbidity and mortality rates in Australia and New Zealand would be similar to those found in Melbourne [2]. In fact, so many similarities were found between the original Melbourne dataset and the regional dataset that it was decided the sets should be pooled to enhance the precision of the regression analysis.

The study focused on elderly non-cardiac major surgery patients aged 70 or above. Major surgery was defined as requiring at least one night’s stay in hospital and generally excluded endoscopy and cataract surgery. The preoperative measures were comorbidity and type of surgery. Postoperative data on complications were collected prospectively and were defined by the REASON team. Patients received a follow-up after 30 days, either in or out of hospital. The primary end-point aimed to identify independent factors for 30-day mortality. Although the analysis used adjusted odds ratios, the focus was largely clinical, looking at patient factors, operative factors and complications. Each dataset collected was adjusted for the preceding one.

### Findings

Twenty percent of the patients from the combined dataset experienced complications within 5 days; 10% were admitted to critical care, of which around 50% were admitted within 5 days, half were admitted electively and the rest were urgent or emergency admissions. Five percent of the patients died within 30 days. There were around 30 complications per 100 patients, with many developing more than one defined complication. On average, patients who experienced one or more complications stayed a week longer in hospital.

### Results by specialty

Looking at the surgical specialties gynecology was shown to have the least frequent mortality and thoracic surgery the most frequent. Multi-trauma surgery also had high mortality. The REASON data were consistent with data from National Surgery Quality Improvement Program (NSQIP) in the United States showing that thoracic surgery is particularly associated with adverse outcomes [3].

Two thirds of the patients in the REASON study were ASA 3 or 4 (50% ASA 3 and 13% ASA 4). The study was also consistent with NSQIP’s suggestion of a curvy linear relationship between mortality and preoperative albumin concentration [4]. The REASON study used an inflection point of 30 g/L to define hypoalbuminaemia that affected 17% of patients and was associated with significantly increased 30-day mortality.

When the odds ratios for mortality were adjusted for patient factors, the odds ratio for mortality in specialty surgery compared to general surgeries fell considerably, particularly in orthopedics, urology and plastics. Even when adjusting for patient factors, thoracic surgery still had by far the strongest association with mortality. These results highlight the importance of taking patient factors into account when looking at mortality rates; as patients become older and sicker and surgery becomes safer, patient factors are increasingly more important than the type of surgery. Preoperative factors and their relationship with independent predictors of mortality were also ranked, with ASA 4 found to be the most important factor in this relationship. The ASA scores stood out statistically over a number of individual comorbidities; both dialysis dependent renal failure and cardiac failure make a patient ASA 4.

### Complications

Complications were ranked by frequency, with the most frequent found to be systemic inflammation and acute renal impairment. The definition used for mild systemic inflammation was SIRS, i.e., inflammation without clear infection. The scale for systemic inflammation ranged, however, from SIRS to septic shock. The definition used for acute renal impairment was 20% increase in creatinine. Patients with these types of complications included those at the more benign end of the disease spectrum, and yet they were associated with marked increases in risk of mortality. These patients at the more benign end are likely to receive less attention in most surgical units, despite the strong associational mortality.

Unplanned ICU admission was also a frequent and important independent predictor of mortality. Like preoperative ASA, unplanned ICU admissions are a reliable indicator of the level of postoperative care within an institution. A paper published in Anesthesiology by Haller et al. supported the theory that unplanned ICU is an indicator of overall quality of postoperative care [5].

As the safety of surgery has improved and rates of surgical mortality and risk of anesthesia have decreased, patient factors have risen. Therefore patient factors have become far more important in risk assessment. ASA status and low albumin are associated with wound infections, which is a useful risk to communicate to orthopedic surgeons in particular, as complications are associated with longer hospital stays.

### Progressive risk assessment

REASON was able to identify certain preoperative variables which were particularly important in terms of patient factors: age, ASA, albumin, urgent surgery and emergency surgery. However, the relevant patient risk increases in the presence of acute renal impairment, even minor inflammation, and unplanned ICU. When a patient has an unplanned ICU admission, their risk assessment is changed; for example, a patient who is relatively well undergoing obesity surgery would find that their risk assessment would be very different if they then ended up in ICU after a surgical complication. It is important to consider how the potential changes in risk assessment should be discussed with the patient.

### Long-term outcome

An important study by NSQIP looked at long-term outcome up to 5 years post-surgery [3]. Patients who experienced either renal failure or systemic sepsis had increased mortality at 30 days, and even after 1 year and 5 years. This emphasizes that these types of events around the perioperative period can have a significant long-term effect.

### Frailty

The REASON study did not consider frailty as a factor in predicting outcome. An NCPOD report on the elderly [6] discussed assessing frailty, suggesting that this is another factor which should be taken into consideration when dealing with elderly patients. Although frailty may be hard to define, some suggested indicators are weight loss, exhaustion, slow walking speed and low physical activity. There may be an inverse relationship between frailty and anaerobic threshold, or VO_2_ max for a given patient, which is an important area that requires further research.

## Conclusions

The REASON study found that patients over 70 could be expected to stay at least one night in hospital; 1 in 5 will have a major complication within 5 days; 1 in 20 will die by day 30; and 1 in 10 will require critical care services, of which half will be unplanned.

Although ASA is deliberately excluded from the P-POSSUM scoring system, the REASON data and previous data from NSQIP support ASA as a good overall preoperative measure. Measuring albumin is a simple procedure which gives an important marker of risk and should be performed more frequently. However, what can be done about risk from albumin in terms of nutrition and chronic disease is unclear at this point. Even mild changes in creatinine or renal function are important and must be closely monitored. Mild inflammation and frailty are also important factors to consider in outcome measurement. Frailty needs to be quantified so that it can be considered as a factor.

A paper by Moore in 2010 [7] noted that sepsis is a far more common surgical complication and is associated with many more deaths than inflammation and pulmonary embolism, and yet receives far less attention in the literature. There is emerging data on the value of laparoscopic surgery.

No one is currently qualified to perform postoperative care exclusively. There are 5 domains in postoperative care: surgical site management; general medicine in the postoperative period; pain medicine; resuscitation; and rehabilitation. Anesthetists can look at where they could improve their understanding in any or each of these areas and also where others could improve their training. It is also important to consider whether single intervention or bundle of care is the best model for a trial. The disadvantage of doing a bundle of care trial, e.g., incorporating nutrition, exercise and postoperative care, is that it is difficult to determine which element of care is making the difference. Therefore with a bundle of care trial it is difficult to isolate specific care conditions in order to do accurate cost-benefit analyses.

## Abbreviations

ASA: American Society of Anesthesiologists; NSQIP: National Surgery Quality Improvement Program; NCPOD: National Confidential Enquiry into Patient Outcome and Death; P-POSSUM: Portsmouth-Physiological and Operative Severity Score for enUmeration of Mortality and morbidity; REASON: Research into Elderly Patient Anesthesia and Surgery Outcome Numbers; SIRS: Systemic inflammatory response syndrome.

## Competing interests

The author has no competing interests.
